# Activation of plasmacytoid dendritic cells promotes AML-cell fratricide

**DOI:** 10.18632/oncotarget.27949

**Published:** 2021-04-27

**Authors:** Kavin Fatehchand, Payal Mehta, Christopher B. Colvin, Nathaniel J. Buteyn, Ramasamy Santhanam, Giovanna Merchand-Reyes, Hafza Inshaar, Brenda Shen, Xiaokui Mo, Bethany Mundy-Bosse, Susheela Tridandapani, Jonathan P. Butchar

**Affiliations:** ^1^Medical Scientist Training Program, Wexner Medical Center, The Ohio State University, Columbus, OH 43210, USA; ^2^Department of Internal Medicine, Wexner Medical Center, The Ohio State University, Columbus, OH 43210, USA; ^3^Center for Biostatistics, Department of Biomedical Informatics, The Ohio State University, Columbus, OH 43210, USA; ^4^Molecular, Cellular and Developmental Biology Program, The Ohio State University, Columbus, OH 43210, USA

**Keywords:** plasmacytoid dendritic cells, interferon-beta, acute myeloid leukemia, fratricide

## Abstract

Acute myeloid leukemia (AML) is characterized by the proliferation of immature myeloid blasts and a suppressed immune state. Interferons have been previously shown to aid in the clearance of AML cells. Type I interferons are produced primarily by plasmacytoid dendritic cells (pDCs). However, these cells exist in a quiescent state in AML. Because pDCs express TLR 7–9, we hypothesized that the TLR7/8 agonist R848 would be able to reprogram them toward a more active, IFN-producing phenotype. Consistent with this notion, we found that R848-treated pDCs from patients produced significantly elevated levels of IFNβ. In addition, they showed increased expression of the immune-stimulatory receptor CD40. We next tested whether IFNβ would influence antibody-mediated fratricide among AML cells, as our recent work showed that AML cells could undergo cell-to cell killing in the presence of the CD38 antibody daratumumab. We found that IFNβ treatment led to a significant, IRF9-dependent increase in CD38 expression and a subsequent increase in daratumumab-mediated cytotoxicity and decreased colony formation. These findings suggest that the tolerogenic phenotype of pDCs in AML can be reversed, and also demonstrate a possible means of enhancing endogenous Type I IFN production that would promote daratumumab-mediated clearance of AML cells.

## INTRODUCTION

Acute Myeloid Leukemia (AML) is associated with defective innate and adaptive immune responses, as is seen with other malignancies. This stems from both direct and indirect interactions with leukemia stem cells (LSCs), regulatory *T*-cells, tolerogenic myeloid stem cells or tolerogenic dendritic cells [1]. Despite this immune suppression, antibody-based immune therapies for AML are being actively explored, either as single agents or in combination with other therapies [2]. In a recent study we demonstrated that daratumumab, a CD38 antibody was able to effectively mediate AML cell-to-cell killing, or fratricide [3]. However, the effect of daratumumab depended on the co-administration of IFNγ to enhance the expression of CD38 and the high affinity IgG receptor CD64/FcγRI. Type I and Type II interferons have previously been tested in clinical trials for AML. Results from a trial with IFNγ showed that it was toxic at dosages equivalent to those needed to induce differentiation of AML cells *in vitro* [4]. Interferon-α (IFNα) has been tested as a maintenance therapy, showing lower toxicity than IFNγ but not conferring any benefit against AML [5, 6]. However, Type I interferons have not been examined as a dual-treatment with anti-CD38 within the context of AML. Type I and Type II interferons signal through distinct pathways but there is also sufficient overlap (e.g., STAT1 phosphorylation) [7] to suggest that they may be of benefit when combined with daratumumab, and with fewer potential toxicities. Among the Type I interferons, IFNβ shows the strongest receptor binding and elicits the most downstream intracellular activity [8].

Plasmacytoid dendritic cells (pDC) are major contributors to Type I IFN responses [9, 10] and also play an important role in bridging innate and adaptive immunity [9]. pDCs express TLR 7-9 and are able to produce many cytokines including TNF-α, CXCL8, and IL-6, but most importantly Type I Interferons after TLR stimulation [11–15]. Within the context of cancer, pDCs have been shown to take on a more tolerogenic state resulting in the induction of regulatory *T*-cells and production of lower amounts of Type I Interferon which is required for tumor clearance [16].

In AML, it has been shown that both FLT3-ITD^+^ and FLT3-ITD^˗^ patients have higher frequencies of circulating pDCs compared to healthy donors. However, these pDCs express lower levels of HLA-DR, which may explain their inability to present antigen as effectively as healthy-donor cells [17]. Nevertheless, since pDCs express Toll-like receptors (TLR) 7–9 [12, 18], activating them with TLR agonists may facilitate a reprogramming toward a more active phenotype [16].

Here, we examined the effects of the TLR7/8 agonist R848 on the phenotype and cytokine production of AML-patient pDCs. In agreement with previous studies [19] we found that R848 led to higher expression of CD40. Notably, it also increased IFNβ production by AML-patient pDCs, and this induced an upregulation of CD38 in AML cells. Consequently, IFNβ combined with the anti-CD38 antibody daratumumab, led to fratricide of the AML cells. Taken together, these results demonstrate that targeting pDCs therapeutically may be an effective means of clearing AML, and this could be particularly useful in patients unable to undergo chemotherapy.

## RESULTS

### TLR 7/8 agonists reverse the tolerogenic phenotype of AML-patient pDCs

**Figure 1:**
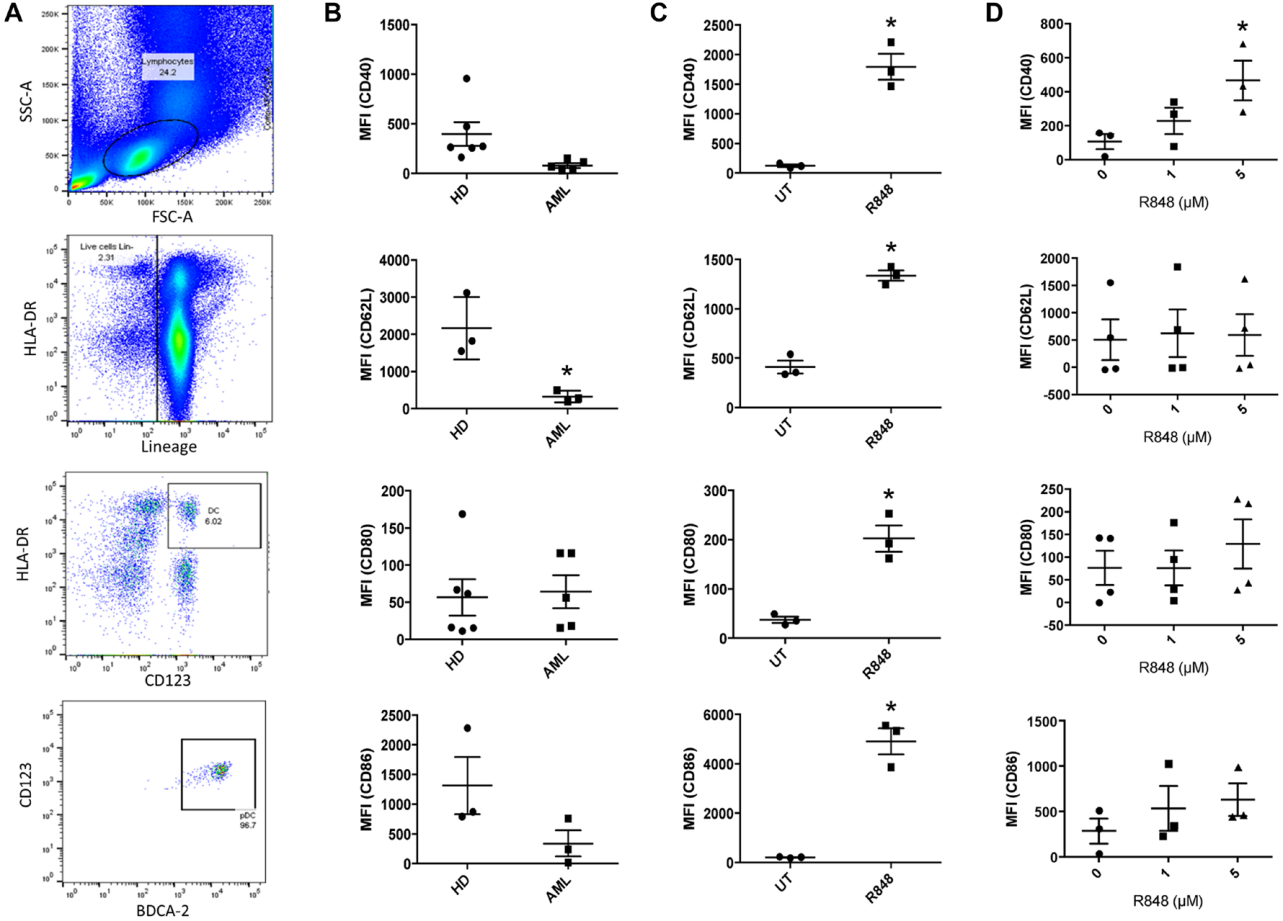
TLR7/8 agonists reverse the tolerogenic phenotype of AML-patient pDCs. (**A**) Identification of pDCs by flow cytometry, gating on scatter, lineage, HLA-DR, CD123 and BDCA2. Exclusion (not shown) was done based on CD3, CD14, CD16, CD19, CD20 and CD56. pDCs were identified as HLA-DR^+^/CD123^+^ followed by BDCA2^+^/CD123^+^. (**B**) Healthy-donor (*n* ≥ 3 donors) and AML-patient (*n* ≥ 3 donors) PBMC were stained in order to identify pDC populations by cytometry described above. Surface expression of CD40, CD62L, CD80 and CD86 on pDCs were measured by flow cytometry. Mean fluorescence intensity (MFI) is plotted. (**C**) Healthy-donor (*n* = 3 donors) PBMC were treated with the TLR7/8 agonist R848 (1 μM) for 24 hours. pDC populations were identified as described above and surface expression of CD40, CD62L, CD80 and CD86 was measured. (**D**) AML-patient (*n* ≥ 3 donors) PBMC were treated with 0, 1 or 5 μM R848 for 48 hours. CD40, CD62L, CD80 and CD86 expression was measured in pDCs as above. ^*^ *p* ≤ 0.05.

Relative to healthy donors, AML patients have elevated frequencies of pDCs, but these cells show decreased expression of activation markers [17]. In order to test whether the TLR7/8 agonist R848 could reverse this, we began by first identifying pDCs from AML patients and from healthy donor PBMCs (Lin^˗^/HLA-DR^+^/CD123^+^/BDCA2^+^, Figure 1A). Next, we measured surface expression of the pDC activation marker CD40 and the transmigration marker CD62L, as well as of CD80 and CD86. As shown in Figure 1B, pDCs isolated from AML patients trended toward reduced CD40 (*p* = 0.07), and CD62L was significantly lower (*p* = 0.02). CD80 and CD86 showed no differences. We next tested whether we could shift the phenotype of pDCs into a more active state. Because pDCs express TLR7-9 [12, 18] we treated PBMCs from healthy donors and from AML patients with the TLR 7/8 agonist R848. Following this we measured surface expression of CD40, CD62L, CD80 and CD86 on the pDCs. As shown in Figure 1C, healthy-donor pDCs responded to 1 μM of R848 within 24 hours (Figure 1C), with significant upregulation of costimulatory markers CD40, CD80 and CD86. This is in agreement with results from Gibson et al. [20]. The migration marker CD62L was also significantly upregulated (Figure 1C). However, AML-patient pDCs showed a more muted response. There was no increase in CD86, CD80 or CD62L. Rather, only CD40 showed an upregulation, and only after 48 hours of incubation with 5 μM R848 (Figure 1D). This exemplifies the resistance of patient pDCs to immune-activating stimuli relative to their healthy-donor counterparts. The upregulation of CD40, however, suggests that they are at least partially responsive to TLR7/8 agonists as CD40 is a driver of activation and maturation [21].

### R848 induces functional changes in AML-patient pDCs

**Figure 2:**
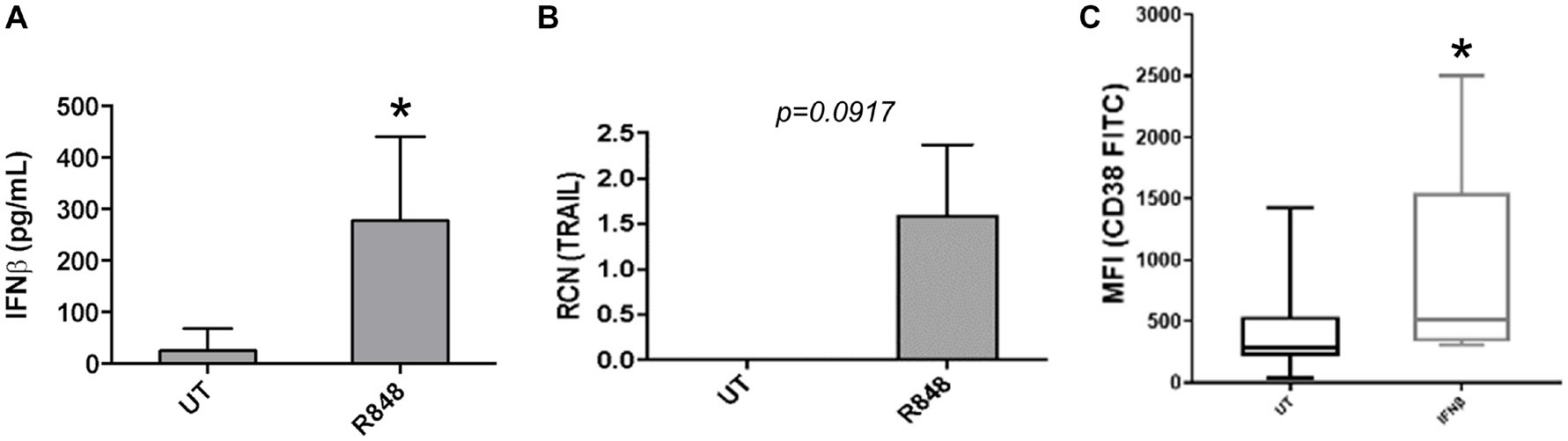
R848 induces functional changes in AML-patient pDCs. (**A** and **B**) pDCs were sorted from AML-patient PBMC samples, then treated with or without R848 (5 μM) for 24 hours. ELISAs were done to measure IFNβ (*n* = 4 donors) (A) and qPCR was done to measure TRAIL (*n* = 7 donors). (B and C) Primary AML apheresis samples (*n* = 8 donors) were treated with or without IFNβ (500 U/mL) and surface expression of CD38 was measured by flow cytometry. Mean fluorescence intensity is plotted. ^*^ *p* ≤ 0.05.

It has been shown that TLR7 agonist treatment can induce pDCs to produce Type I IFNs [22, 23], which drives antitumor effects. Along with this they may effect cytotoxicity via TRAIL [22, 23]. To confirm the ability of the TLR7/8 agonist R848 to similarly activate pDCs, we FACS-sorted AML-patient pDCs and treated them for 48 hours with or without R848. IFNβ and TRAIL were measured by ELISA and qPCR, respectively. Results showed that R848-treated pDCs expressed significantly higher levels of IFNβ (Figure 2A, *n* = 4 donors). TRAIL expression was substantially elevated, but the difference was not statistically significant with a two-tailed *t-test* (Figure 2B, *n* = 7 donors, *p* = 0.0917).

Although TRAIL has previously been shown to be induced in pDCs [22, 23], the stronger effect of R848 on IFNβ as well as the known insensitivity of AML cells to TRAIL-mediated killing [24–26] led us to focus on IFNβ. In particular, we investigated whether pDC-derived IFNβ may lead to upregulation of a targetable antigen on AML cells. We had previously shown that the Type II interferon IFNγ led to strong upregulation of CD38 on AML cells, which subsequently facilitated cytotoxicity of AML cells amongst themselves upon addition of the anti-CD38 antibody daratumumab [3]. Hence, we sought to determine whether IFNβ acted in a similar manner, up-regulating CD38 on AML cells. For this, primary AML apheresis samples were treated for 24 hours with or without IFNβ and surface expression of CD38 was measured by flow cytometry. Results showed that IFNβ significantly increased the expression of CD38 (Figure 2C, *n* = 8 donors).

### IFNβ-mediated up-regulation of CD38 requires canonical and non-canonical pathways

We have previously shown that Type II IFN upregulated CD38 on AML cells through p38, NF-κB, and JAK/STAT [3]. Type I and Type II IFN pathways differ in some downstream signaling components [27], so we sought to determine the mechanism by which IFNβ up-regulated CD38. In the canonical pathway, Type 1 IFNs bind to the IFNAR receptor, which consists of the IFNAR1 and IFNAR2 subunits. IFNAR1 associates with the Jak-family kinase Tyk2, while IFNAR2 associates with Jak1. Ligand binding leads to phosphorylation of Jak1 and Tyk2. Following this, STAT1 and STAT2 are phosphorylated, leading to their activation. This results in the formation of the IFN-stimulated gene factor 3 (ISGF3) complex as IRF9 is recruited to the phosphorylated STAT1/STAT2 dimer. This then induces expression of Interferon Stimulated Genes (ISGs) [27]. In the non-canonical pathway, Type 1 Interferons can act independently of STAT signaling, through both the PI3K and MAPK pathways [28]. Erk can also play a role apart from ISGF3, associating with STAT1 following IFNβ treatment [29].

**Figure 3:**
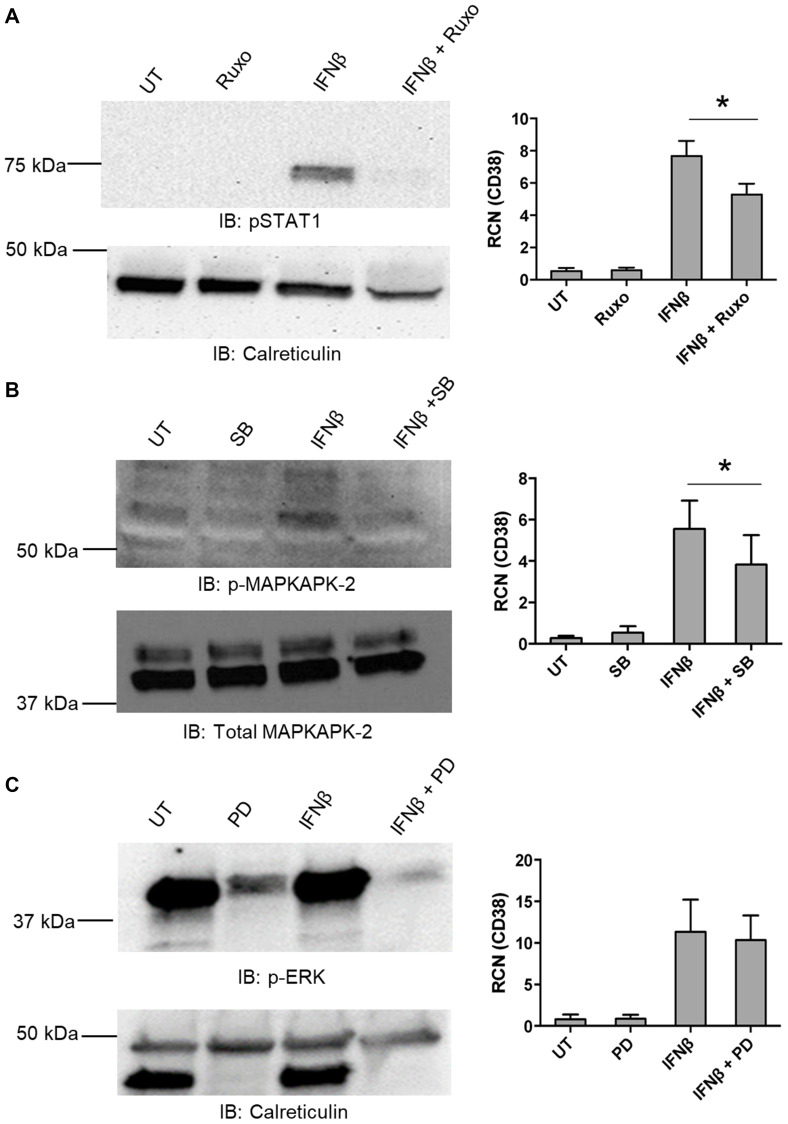
IFNβ-mediated up-regulation of CD38 requires canonical and non-canonical pathways. (**A**–**C**) Primary AML apheresis samples were pre-treated for 30 minutes with either the JAK 1/2 inhibitor Ruxolitinib (Ruxo, 5 nM, *n* = 4 donors) (A), the p38 inhibitor SB202190 (SB, 1 μM, *n* = 6 donors) (B) or the Mek inhibitor PD0325901 (PD, 0.5 μM, *n* = 5 donors) (C) and then treated for 24 hours with or without IFNβ (500 U/mL). For each respective set, qPCR was done to measure CD38 (A–C, right-hand panels). Left-hand panels show representative blots verifying the efficacy of the inhibitors. ^*^ *p* ≤ 0.05.

To determine which of these were required for IFNβ-mediated up-regulation of CD38 expression, primary AML apheresis samples were pre-treated with inhibitors for Jak 1/2 (Ruxolitinib, targeting the JAK/STAT canonical pathway), p38 (SB202190, targeting the non-canonical p38 MAPK pathway) and MEK (PD0325901, targeting Erk). Western blots were done to verify the efficacy of the Jak 1/2 inhibitor (Figure 3A, left panel), the p38 inhibitor (Figure 3B, left panel) and Erk inhibitor (Figure 3C, left panel). qPCR results showed that upregulation of CD38 by IFNβ was significantly dampened by the inhibition of both Jak 1/2 and p38 (Figure 3A and 3B, right panels). MEK/ERK did not seem to be required for this IFNβ-mediated up-regulation of CD38 (Figure 3C, right panel). As shown in the blot (Figure 3C, left upper panel), we observed strong basal Erk phosphorylation. This is frequently seen in AML samples, both cell-line and primary [30–32]. However, the efficacy of PD0325901 was seen, as it substantially reduced Erk phosphorylation.

These results indicate that both the canonical and non-canonical pathways play a role in IFNβ-mediated up-regulation of CD38 in AML cells. It should be noted that the IC_50_ of Ruxolitinib for Tyk2 is approximately fourfold of what was used here (19 nM ± 3.2 [33] versus our use of 5 nM). Tyk2 has been shown to drive activation of Vav [34], which leads to Rac1 activation and this is required for IFN-mediated activation of p38 (reviewed in [27, 35]). It has also been shown that inhibition of p38 does not interfere with STAT1/STAT2 phosphorylation, nor formation of the canonical ISGF3 complex consisting of STAT1, STAT2 and IRF9 [36].

### pDC-dependent IFNβ production enhances CD38 expression on AML cells

**Figure 4:**
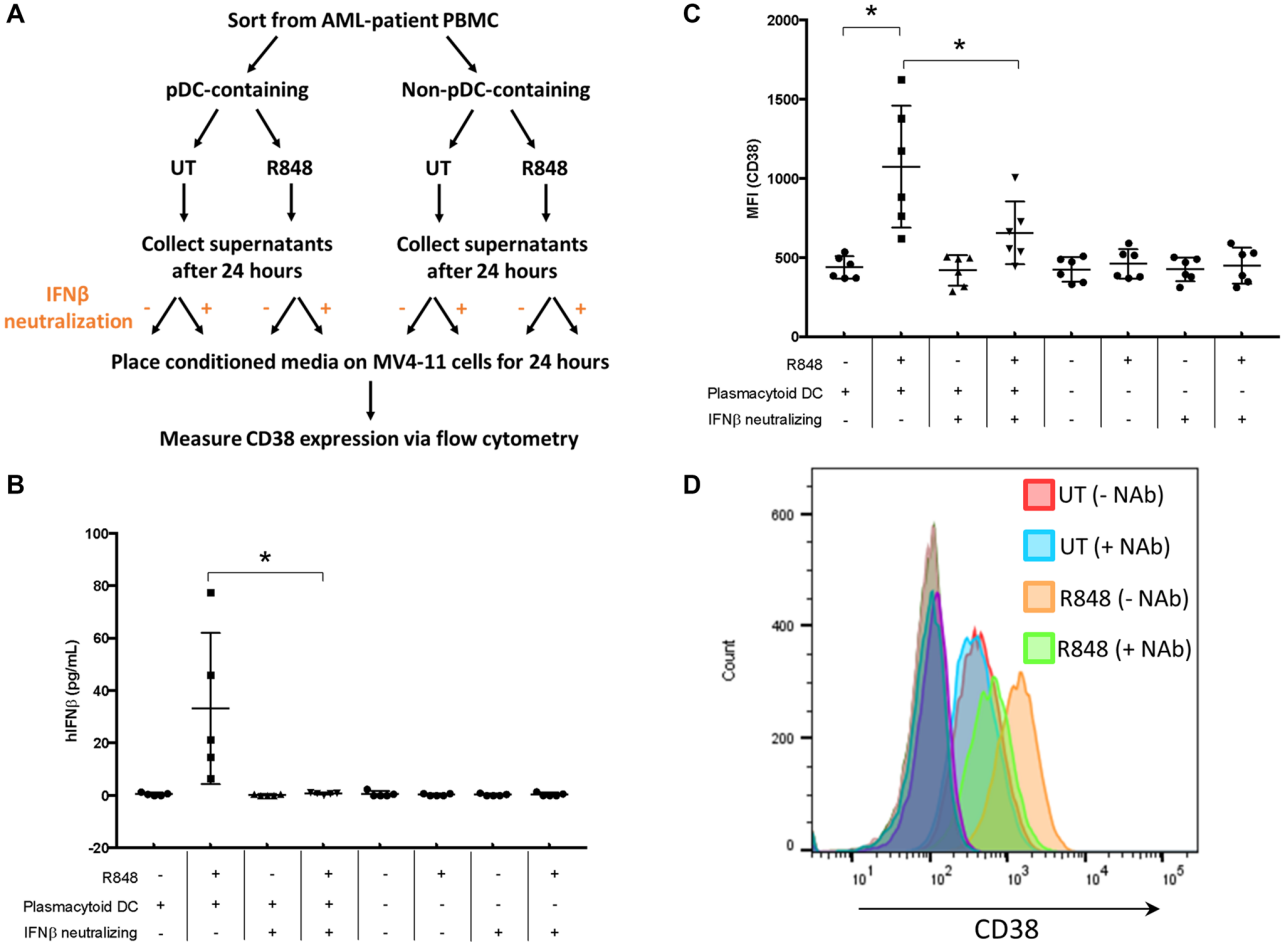
pDC-dependent IFNβ production enhances CD38 expression on AML cells. (**A**) Schematic of experimental procedure. AML PBMC samples (*n* = 5) were sorted for both pDC^+^ and pDC^–^ populations, then treated overnight with or without R848 (5 μM). Supernatants were collected and placed on MV4-11 cells in the presence or absence of IFNβ-neutralizing antibody (1000 U/mL) for 24 hours. (**B**) Supernatants were collected and IFNβ measured by ELISA. (**C**) Cells were stained for CD38 and surface expression measured by flow cytometry. (**D**) Representative histogram from (C). ^*^ *p* ≤ 0.05.

Having determined the molecular mechanism by which recombinant IFNβ treatment results in enhanced CD38 expression on AML cells, we next asked whether pDC-dependent IFNβ production after R848 treatment could enhance CD38 expression on AML cells in a paracrine fashion. For this, we collected pDC-positive and pDC-negative fractions by flow-cytometry sorting of primary AML PBMC samples. The two sets of primary AML cells, pDC-positive and pDC-negative, were then treated with R848 (5 μM) overnight. Cell supernatants from both sets were collected and placed on MV4-11 cells in the presence or absence of a neutralizing antibody against IFNβ for 24 hours. A schematic for this primary-cell treatment and subsequent conditioned-media assay is shown in Figure 4A. Supernatants from the primary cells treated with R848 were collected to measure IFNβ by ELISA (Figure 4B, *n* = 5 donors). Results showed that R848 enhanced IFNβ production in the pDC-positive but not pDC-negative samples (Figure 4B), suggesting that this IFNβ response was more rapid than the required 48 hours for CD40 upregulation as in Figure 1. As expected, this increased IFNβ was offset by IFNβ-neutralizing antibody (Figure 4B). Conditioned media from these primary-cell cultures was then placed onto MV4-11 cells for 24 hours. Following this, CD38 expression on the MV4-11 cells was measured using flow cytometry. Results showed that CD38 expression was upregulated in MV4-11 cells that had been incubated with the conditioned media from pDC-positive cultures that had been treated with R848. This was significantly attenuated by IFNβ-neutralizing antibody (Figure 4C and 4D). In MV4-11 cells treated with conditioned media from pDC-negative cells, there was no upregulation of CD38, even if the conditioned media contained R848. Hence, there was no direct effect on AML cells from R848 in the conditioned media with respect to CD38 expression. These results suggest that R848 elicits IFNβ from pDCs, and that this IFNβ is biologically active and leads to the upregulation of CD38 on AML cells.

### IFNβ-induced AML-cell cytotoxicity is enhanced with anti-CD38 antibody daratumumab

**Figure 5:**
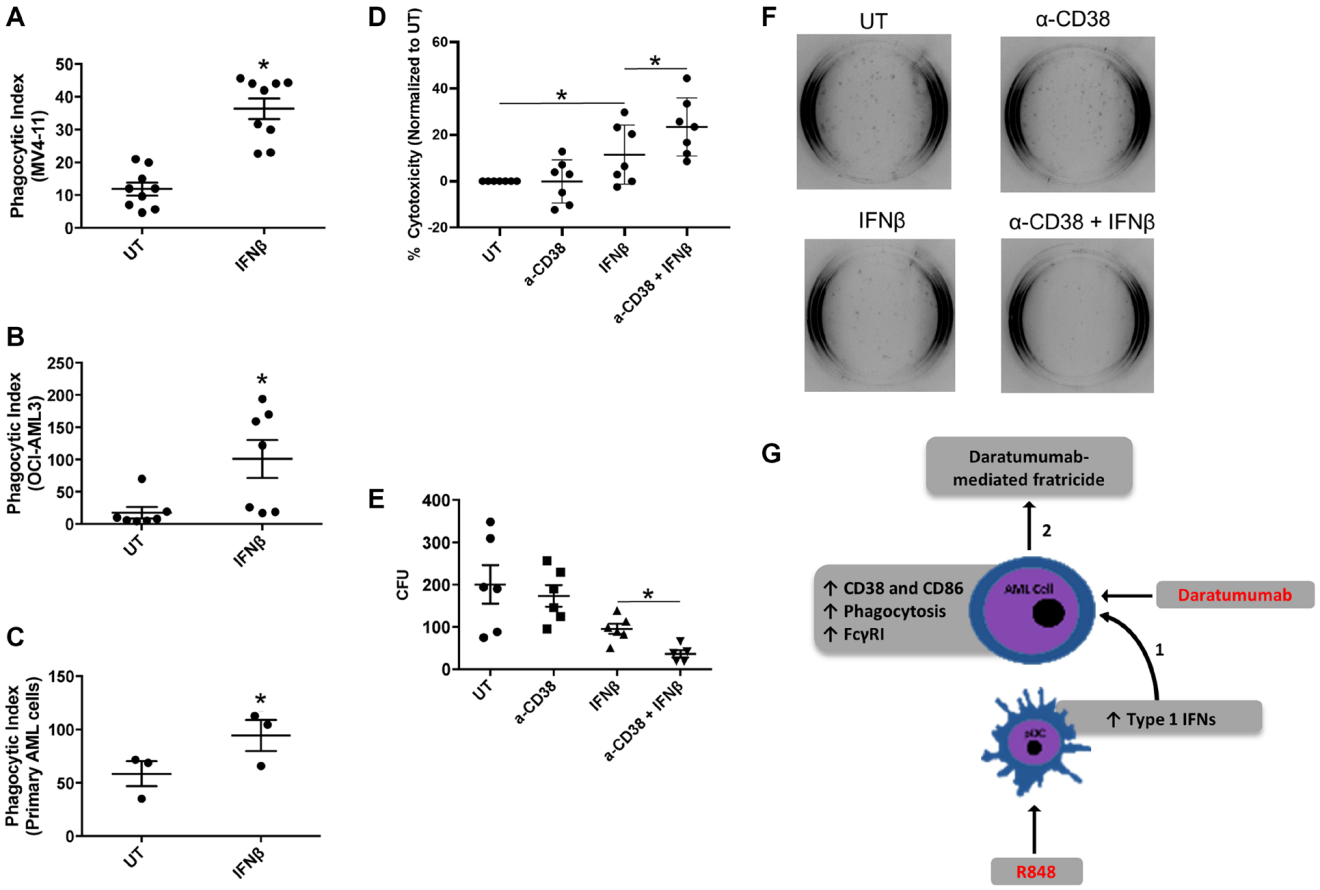
IFNβ-induced AML-cell cytotoxicity is enhanced with anti-CD38 antibody daratumumab. (**A**–**C**) AML cell lines (MV-411, *n* = 9 experiments and OCI-AML3, *n* = 7 experiments) and primary AML apheresis samples (*n* = 3) were treated with or without 1000 U/mL IFNβ for 24 h and then incubated with opsonized sheep red blood cells. Phagocytosis was evaluated via microscopy in a blinded fashion. The phagocytic index represents the number of red blood cells ingested by 100 AML cells for each respective cell line. (**D**) OCI-AML3 cells (*n* = 7 experiments) were treated with or without anti-CD38 antibody (α-CD38, 20 μg/mL), IFNβ (500 U/mL), or α-CD38 + IFNβ for 48 hours. Cytotoxicity was then measured using a Lactate Dehydrogenase Assay. (**E** and **F**) MV4-11 cells (*n* = 6 experiments) were treated with IFNβ (500 U/mL) for 48 hours. Cells were then plated on methocult™-media-containing plates for 10 days, then colonies counted in a double-blinded fashion. (**G**) Current working model, as described in the text. ^*^ *p* ≤ 0.05.

We next examined whether IFNβ could induce functional changes in AML cells, such as those seen earlier with IFNγ [3]. We treated two AML cell lines (OCI-AML3 and MV4-11) and primary AML apheresis samples with IFNβ (500 U/mL) for 24 hours and tested their ability to phagocytose antibody-opsonized target cells, here opsonized sheep red blood cells. As shown in Figure 5A–5C, IFNβ treatment significantly enhanced the phagocytic capacity of both cell lines (Figure 5A and 5B) and primary AML cells (Figure 5C).

We next tested the ability of IFNβ to promote antibody-mediated fratricide among AML cells. Here, we treated the AML cell line OCI-AML3 with or without IFNβ (500 U/mL) in the presence or absence of the anti-CD38 antibody daratumumab (20 μg/mL) for 48 hours. Cell death was measured via lactate dehydrogenase (LDH) release. Results indicated that IFNβ alone significantly increased cytotoxicity (Figure 5D), which was in line with our previous findings with IFNγ [3]. Notably, the combination of IFNβ and daratumumab led to significantly higher cytotoxicity than IFNβ alone (Figure 5D). Because this assay was done using conditioned media and an AML cell line rather than primary samples containing multiple cell types, these results suggest that daratumumab may have promoted cell-against-cell fratricide. We have observed such fratricide in an earlier study using daratumumab [3].

In order to corroborate these findings, we treated the AML cell line MV4-11 with or without IFNβ (500 U/mL) in the presence or absence of daratumumab for 48 hours. We then performed a 10-day colony-forming assay, scoring colony-forming units in a double-blinded fashion. Results showed that IFNβ trended toward a reduced number of colonies (*p* = 0.09), but the combination of IFNβ plus daratumumab led to significantly fewer colonies than IFNβ alone (Figure 5E and 5F). Taken together, these results suggest that activation of TLR7/8 on pDCs induces the production of IFNβ, which then provides an opportunity for daratumumab-mediated AML-cell fratricide.

## DISCUSSION

pDCs have been shown to be the major cell type for Type I interferon production ([20, 37] and reviewed in [38] and [39]). Here, we have shown that activation of AML-patient pDCs through TLR 7/8 agonist treatment ultimately leads to AML-cell destruction by IFNβ and daratumumab. Combined IFNβ and daratumumab led to significantly greater cell death than IFNβ alone, suggesting that daratumumab may have promoted AML-cell fratricide. We have observed this phenomenon previously in a study of Type II interferon [3]. R848-treated pDCs showed higher expression of CD40, and produced IFNβ. IFNβ treatment of AML cells led to increased expression of CD38, and conferred the ability to target one another for fratricide. In our working model (Figure 5G), we propose that R848 can activate pDCs such that they produce IFNβ, which in turn increases expression of the daratumumab target CD38 on AML cells. This leads to antibody-mediated AML-cell fratricide.

By examining the activation and costimulatory markers CD40, CD80 and CD86 as well as the migration marker CD62L, we were able to show that the AML-patient pDCs express less CD62L (Figure 1B) and that they are less responsive in general to R848 than healthy-donor pDCs (Figure 1D versus Figure 1C). The responses to R848 seen with the healthy-donor cells are in agreement with Gibson et al., 2002 [20]. Even though R848 did not activate patient-derived pDCs to the same degree, it did lead to upregulation of CD40 and production of IFNβ. This is important, as CD40 activity drives activation and maturation [21] and its expression is often reduced in tumor-associated dendritic cells [40]. It also suggests that these cells retain the ability to be at least partially responsive to immune modulators such as TLR7/8 agonists. CD62L, a marker of motility [41], is strongly expressed on pDCs [42] yet reduced in pDCs within the context of cancers such as non-Hodgkin’s lymphoma [43]. Our findings are in agreement, such that pDCs from AML patients express significantly less CD62L than those from healthy donors.

pDCs can contribute to tumor clearance by inducing TRAIL-mediated apoptosis or by the production of Type 1 Interferons [23, 44]. R848 did induce some TRAIL expression on pDCs from the AML-patient samples we tested (*p* = 0.09), but AML cells are relatively insensitive to TRAIL. This phenomenon has previously been reported but certain agents have been shown to sensitize AML cells to TRAIL-dependent killing. For example, triptolide is able to sensitize AML cells to TRAIL-mediated apoptosis by decreasing XIAP expression and increasing DR5 expression on AML cells [25]. However, due to its severe toxicity and water-insolubility, the clinical use of this drug is quite limited [45]. Nonetheless, newer water-soluble pro-drugs, such as Minnelide, have been developed which have entered Phase II clinical trials (NCT03117920) [46]. In combination with R848 or similar TLR agonists, Minnelide may help induce TRAIL-mediated apoptosis of AML cells introducing another possible therapeutic strategy for AML.

R848 is also able to significantly upregulate the surface expression of CD40 on AML-patient pDCs. Although pDCs take up less antigen than myeloid dendritic cells, they have been shown to induce potent CD4^+^ and CD8^+^ T cell responses when activated [47]. Activated pDCs express high levels of the co-stimulatory molecule CD86 (B7–2) which, when bound to CD28 on T-cells can induce T cell activation [48]. Another important interaction is between CD40 on pDCs and CD40L on T cells. Upon this interaction, IL-6 is released by pDCs, which then allows B cells to become antibody secreting plasma cells [48–52]. These R848-induced phenotypic changes help shift pDCs into an activated state, which can then potentially play a role in anti-tumor immunity both directly and indirectly.

Although we did not see clear patterns between mutational status/cytogenetic profile and the ability of the pDCs to produce IFNβ, we did see that it was only the monocytic-lineage group (M5 classification by FAB) that showed this response. Further studies are required with larger sample numbers to determine more precisely which subtypes of AML are associated with IFNβ-producing pDCs. For example, there is a possibility that pDCs express different levels of molecules such as ILT7 [53], which recognizes Bone Marrow Stromal Antigen 2 (BST2) that is expressed on tumor cell lines and primary carcinomas [54, 55]. Binding of ILT7 to BST2 may decrease Type I Interferon production [16]. Perhaps disrupting this ILT7/BST2 interaction could lead to enhanced IFNβ production in all AML subtypes.

Both the Type I IFNs, IFNα and IFNβ have been approved by the FDA for clinical use. IFNα has been used in the treatment of hairy cell leukemia, chronic myeloid leukemia, and has been studied in the context of AML [56–58]. In the setting of AML, IFNα therapy has been tested in multiple clinical trials in the context of inducing remission, salvage therapy, and post-remission therapy [59–61]. Type I IFNs have both direct and indirect effects on AML cells, which provides rationale for their use. Type I IFNs are able to induce both the canonical and non-canonical pathways, which can eventually induce apoptosis, inhibit cell proliferation, enhance AML cell immunogenicity, sensitize AML cells to differentiation, and reduce growth-promoting cytokine production by the AML cells [59]. In addition, Type I IFNs can increase the cross-priming ability of dendritic cells, and increase the cytotoxic abilities against leukemic cells by both DC [59, 62, 63], T [59, 64] and NK cells [59]. Although IFNα has shown promising results *in vitro*, there have been problems that have led to modest results in *in vivo* and in clinical trials. Both IFNα and IFNβ have relatively short serum half-lives. Benjamin et al. showed that stable expression of IFNβ by virus-mediated gene transfer resulted in anti-leukemic effects when compared to bolus administration of IFNβ. Although IFNβ levels were as low as 10 IU/mL in mice with stable expression of IFNβ, AML tumor burden was significantly reduced when compared to control treated animals [65]. These data show the importance of generating longer lasting IFN preparations in order to achieve clinical efficacy. Both pegylated IFNs and albumin-IFN fusion proteins have been developed that help extend the serum half-life [66]. Pegylated forms of IFNα and IFNβ have been used in the setting of Hepatitis B and C (Pegylated Alfa-2a/Pegasys, Genentench Inc.) and in Multiple Sclerosis (Pegylated interferon beta-1a/Plegridy, Biogen Canada) [67–69]. Here, we demonstrate another approach to overcome the limitation of the short half-life of exogenous IFNβ by inducing endogenous production of Type I IFN.

In summary, we report a novel mechanism of inducing the effector-like AML cell phenotype by reprogramming AML-patient pDCs to produce IFNβ through TLR stimulation. This can lead to upregulation of CD38 on AML cells and can enhance antibody-mediated fratricide of AML cells. These findings suggest that the use of either IFNβ or IFNβ-inducing agents in combination with an anti-CD38 therapeutic antibody could likely offer a new therapeutic option for AML.

## MATERIALS AND METHODS

### Antibodies and reagents

Recombinant human IFNβ-1a (PBL, Piscataway, NJ, USA) was added to cell cultures at a concentration of 500 U/mL. Daratumumab was used for LDH (20 μg/ml) and colony forming assays. For flow cytometry, unconjugated mouse anti-human CD64 (clone 32.2) with an FITC goat anti-mouse secondary antibody (Invitrogen), anti-human CD38 conjugated to FITC (clone HIT2; BD Biosciences), anti-human CD86 conjugated to phycoerythrin (clone 2331 (FUN-1); BD Biosciences) were used to measure markers of activation on AML cells. For sorting, anti-human Lineage cocktail conjugated to APC (clone UCHT1, HCD14, 3G8, HIB19, 2H7, HCD56, Biolegend), anti-human HLA-DR conjugated to APC/Cy7 (clone L243, Biolegend), anti-human BDCA-2conjugated to FITC (clone 201A, Biolegend), and anti-human CD123 conjugated to PE/Cy7 (clone 6H6, Biolegend) were used. For the identification and measurement of activation status of plasmacytoid dendritic cells, anti-human Lineage cocktail conjugated to BV510 (clone OKT3, M5E2, 3G8, HIB19, 2H7, HCD56, Biolegend), anti-human CD123 conjugated to BV650 (clone 6H6, Biolegend), anti-human HLA-DR conjugated to APC/Cy7 (clone L243, Biolegend), anti-human BDCA-2 conjugated to PE/Cy7 (clone 201A, Biolegend), anti-human CD80 conjugated to BV421 (clone 2D10, Biolegend), anti-human CD62L conjugated to PerCP (clone DREG-56, Biolegend), anti-human CD86 conjugated to APC (clone IT2.2, Biolegend), and anti-human CD40 conjugated to PE (clone 5C3, Biolegend) were used. Samples were analyzed using an LSRII flow cytometer (BD Bioscience) and FlowJo software (FLOWJO, LLC, Ashland, OR, USA). The JAK1/2 inhibitor, ruxolitinib (used at 50 nM), was purchased from Selleck Chemicals (Houston, TX, USA). The MEK inhibitor, PD0325901 (used at 0.5 μM) was purchased from Selleck Chemicals (Houston, TX, USA). The p38 MAPK inhibitor, SB202190 (used at 1 μM) was purchased from Selleck Chemicals (Houston, TX, USA).

### Cell culture

The AML cell lines used in this study (MV4-11 and OCI-AML3) were purchased from ATCC and cultured according to ATCC recommendations. Cells were maintained below 1 × 10^6^ cells/mL in RPMI 1640 media (Gibco, Grand Island, NY, USA) supplemented with 10% heat-inactivated fetal bovine serum (FBS; Hyclone Laboratories, Grand Island, NY, USA), 2 mM L-glutamine (Invitrogen, Grand Island, NY, USA), and penicillin/streptomycin (56 U/mL/56 μg/mL; Invitrogen) at 37°C in an atmosphere of 5% CO_2_.

### Primary cells

Primary cell handling was done as described previously [3]. White blood cells apheresed from AML patients were obtained after written informed consent in accordance with the Declaration of Helsinki under a protocol approved by the institutional review board of The Ohio State University. Cells were stored in liquid nitrogen in 20% FBS and 10% DMSO until needed for experiments. At the time of the experiment, cells were thawed at 37°C and incubated in RPMI 1640 media (Gibco) supplemented with 20% FBS, 2 mM L-glutamine (Invitrogen) and penicillin/streptomycin (56 U/mL/56 μg/mL; Invitrogen) at 37°C in an atmosphere of 5% CO_2_ for 1 hour. Cells were then centrifuged and incubated at 3 × 10^6^/mL in RPMI 1640 media (Gibco) supplemented with 20% FBS, 2 mM L-glutamine (Invitrogen) and penicillin/streptomycin (56 U/mL/56 μg/mL; Invitrogen) and were either left untreated or treated with Interferon-beta (IFN-β) and incubated for 24 hours at 37°C. The next day, cells were counted using Trypan blue exclusion and used for assays.

### Western blotting

Anti-pSTAT1, Anti-p-MAPKAPK-2, Anti-p-ERK, Anti-MAPKAPK-2, and anti-IRF9 for Western blotting were purchased from Cell Signaling Technology (Danvers, MA, USA). Anti-Calreticulin antibody was purchased from Enzo Life Sciences (Farmingdale, NY, USA). Anti-GAPDH antibody was purchased from Santa Cruz Biotechnology (Dallas, TX, USA). Western blotting was done as described previously [70]. Cells were lysed in TN1 buffer (50 mM Tris (pH 8.0), 10 mM EDTA, 10 mM Na_4_P_2_O_7_, 10 mM NaF, 1% Triton X-100, 125 mM NaCl, 10 mM Na_3_VO_4_, and 10 μg/ml each aprotinin and leupeptin). Protein lysates were boiled in Laemmli sample buffer, separated by SDS-PAGE, transferred to nitrocellulose membranes, probed with the antibody of interest, and then developed by Pierce ECL 2 Western blotting substrate (Thermo Scientific, Rockford, IL, USA) or SuperSignal West Femto maximum sensitivity substrate (Thermo Scientific). Densitometry was performed using ImageJ software (National Institutes of Health, Bethesda, MD, USA), and ratios between the indicated probes and their respective anti-actin reprobes were calculated.

### Colony forming assay

MV4-11 cells were treated with or without IFN-β (500 U/mL) in the presence or absence of daratumumab (20 μg/mL, supplied from commercial sources, The Ohio State University, Columbus, OH, USA) for 24 hours then plated in duplicates in Methocult™ H4100 methylcellulose medium (StemCell Technologies) on cell culture plates for 10 days. After 10 days, colonies were scored in a double-blind fashion.

### Lactate dehydrogenase assay

OCI-AML3 cells were plated at 5 × 10^5^ cells/mL and treated with 500 U IFNβ and/or 20 μg/mL daratumumab. After 48 hours, supernatants were removed and used for a CytoTox96^®^ Non-Radio Cytotoxicity Assay (Promega, Madison, WI, USA) according to manufacturer’s instruction. Percent cytotoxicity was defined as [Experimental LDH release OD_490_ / Maximum LDH release OD_490_] × 100.

### Phagocytosis

Phagocytosis assays were performed as described previously with minor adaptations for the experimental requirements of this study [71]. Briefly, sheep red blood cells (SRBCs; Colorado Serum Company, Denver, CO, USA) were labeled with PKH26 fluorescent cell membrane dye (Sigma) and then opsonized with anti-SRBC antibody (Sigma). SRBCs were added to the respective AML cell lines (treated with IFN-β for 24 hours) or primary AML apheresis samples (treated with IFN-β for 24 h), gently pelleted by slow centrifugation, and then incubated at 37°C for 1 hour. Non-phagocytosed SRBCs were lysed with red blood cell lysis buffer (eBioscience, San Diego, CA, USA) at room temperature for 10 min and washed with PBS before fixation with 4% paraformaldehyde. The SRBCs ingested by the AML cells were counted in a blinded fashion using fluorescence microscopy, with three separate such counts per condition. For each set of counts, 100 AML cells per condition were examined. The phagocytic index is defined as the total number of SRBCs ingested by 100 AML cells.

### Real-time PCR

Total RNA was isolated using the Norgen Biotek Total RNA Purification Kit (Norgen Biotek Corp., Ontario, Canada) according to manufacturer’s instructions. RNA was reverse transcribed and subjected to quantitative real-time PCR using Power SYBR Green Master Mix (Applied Biosystems, Grand Island, NY, USA) as previously described [3]. The following primers were used: GAPDH (forward primer, 5ʹ-ATT CCC TGG ATT GTG AAA TAG TC-3′; reverse primer, 5′-ATTAAAGTCACCGCCTTCTGTAG-3′), CD38 (forward primer, 5′-GCTCAATGGATCCCGCAGT-3′; reverse primer, 5′-TCCTGGCARAAGTCTCTGG-3′), and TRAIL (forward primer 5′-AAG GCT CTG GGC CGC AAA ATA AAC-3′and reverse primer 5′-GCC AAC TAA AAA GGC CCC GAA AAA-3′). GAPDH was used for normalization of the genes of interest. Data were presented as mean relative copy number for at least three separate experiments using relative copy number = 2^−Δ*Ct*^ × 100 [72], where Δ*Ct* is the *Ct*_target_ − *Ct*_GAPDH_.

### Statistics

For experiments that involved placing the cells of each donor across multiple conditions, mixed-effect modeling was done. For experiments with only two groups involved, unpaired two-tailed (Figure 1B) or paired two-tailed (all others) Student’s *t-test*s were used to test for statistically significant differences. Analyses were performed using SAS (SAS Inc., Cary, NC, USA) and Microsoft Excel (Microsoft, Redmond, WA, USA).

## Abbreviations

pDC: plasmacytoid dendritic cell
AML: acute myeloid leukemia
IFNβ: Interferon-beta
